# Medical Reciprocity

**Published:** 1903-08

**Authors:** 


### MEDICAL RECIPROCITY.

THE following abstract from an editorial in *Science* indicates that our medical friends are beginning to feel the iron hand of State boards for medical examination, and it is understood that even the legal profession is beginning to revolt. As the world progresses in legal refinement State boards will be instituted for the examination of clergymen, and then we will see the novel performance of a minister being obliged to pass an examination before he can preach the gospel in the several States of this Union. This may seem absurd to some minds, but it is the logical result of this force by law.

"MEDICAL RECIPROCITY BETWEEN THE STATES OF THE UNION.

"The low requirements of some medical colleges, and the want of uniformity in the requirements for a license to practise in the different States, has resulted in a condition which entails much hardship on a physician who desires to remove from one and to engage in practice in another State. The rules of most State boards of medical examination and of health are so stringent that a physician or surgeon of years of experience and of acknowledged skill and education, and the specialist who may be renowned in his field of work, are obliged, like the recent graduate, to take an examination in all of the branches of medicine and surgery in order to secure a license to practise in the State of his adoption.

"To correct this evil it has been suggested by a member of the American Medical Association, and concurred in by others, that a national board of medical examiners be organized; that the board hold examinations at different seasons of the year in the various large cities, and that the diploma so obtained shall be recognized as a license to practise in any one or all of the States and Territories. The measure suggested seems to be practical and feasible."

